# Tracking Stress, Mental Health, and Resilience Factors in Medical Students Before, During, and After a Stress-Inducing Exam Period: Protocol and Proof-of-Principle Analyses for the RESIST Cohort Study

**DOI:** 10.2196/20128

**Published:** 2021-06-08

**Authors:** Jessica Fritz, Jan Stochl, Rogier A Kievit, Anne-Laura van Harmelen, Paul O Wilkinson

**Affiliations:** 1 Department of Psychiatry University of Cambridge Cambridge United Kingdom; 2 Medical Research Council Cognition and Brain Sciences Unit University of Cambridge Cambridge United Kingdom; 3 Radboud University Medical Center, Donders Institute for Brain, Cognition, and Behavior Nijmegen Netherlands; 4 Brain Safety and Resilience, Education and Child Studies, Leiden University Leiden Netherlands

**Keywords:** exam stress, perceived stress, mental distress, student mental health, mental health resilience, protective factors, resilience factors

## Abstract

**Background:**

Knowledge of mental distress and resilience factors over the time span from before to after a stressor is important to be able to leverage the most promising resilience factors and promote mental health at the right time. To shed light on this topic, we designed the RESIST (Resilience Study) study, in which we assessed medical students before, during, and after their yearly exam period. Exam time is generally a period of notable stress among medical students, and it has been suggested that exam time triggers mental distress.

**Objective:**

In this paper, we aim to describe the study protocol and to examine whether the exam period indeed induces higher perceived stress and mental distress. We also aim to explore whether perceived stress and mental distress coevolve in response to exams.

**Methods:**

RESIST is a cohort study in which exam stress functions as a within-subject natural stress manipulation. In this paper, we outline the sample (N=451), procedure, assessed measures (including demographics, perceived stress, mental distress, 13 resilience factors, and adversity), and ethical considerations. Moreover, we conducted a series of latent growth models and bivariate latent change score models to analyze perceived stress and mental distress changes over the 3 time points.

**Results:**

We found that perceived stress and mental distress increased from the time before the exams to the exam period and decreased after the exams to a lower level than before the exams. Our findings further suggest that higher mental distress before exams increased the risk of developing more perceived stress during exams. Higher perceived stress during exams, in turn, increased the risk of experiencing a less successful (or quick) recovery of mental distress after exams.

**Conclusions:**

As expected, the exam period caused a temporary increase in perceived stress and mental distress. Therefore, the RESIST study lends itself well to exploring resilience factors in response to naturally occurring exam stress. Such knowledge will eventually help researchers to find out which resilience factors lend themselves best as prevention targets and which lend themselves best as treatment targets for the mitigation of mental health problems that are triggered or accelerated by natural exam stress. The findings from the RESIST study may therefore inform student support services, mental health services, and resilience theory.

## Introduction

### Background

Approximately 1 in 5 young people experience mental distress in the form of anxiety and depression [1,2]. According to the World Health Organization (ie, the Regional Office for Europe), the “early identification of such problems—and, when necessary, early intervention or timely management—is critically important...In the absence of appropriate support and intervention, such problems may continue, worsen or lead to mental illness” [2]. Resilience factors (RFs), such as self-esteem and friendship support, mitigate mental distress in the face of stressful experiences [3]. The literature contains a considerable amount of knowledge on RFs that mitigate concurrent and subsequent mental distress [4-8]. Yet, studies investigating RFs over the time span from before to after a stressor (ie, stress that causes or triggers mental distress) are scarce [9]. However, knowledge of mental distress and RFs *before* and *during* the stressor is crucial, as this (1) is necessary to determine whether mental distress and RFs are affected by the stressor [9,10], and (2) enables the identification of those RFs that are potentially promising prevention targets. Knowledge on mental distress and RFs *during* and *after* the stressor is equally essential, as this (1) enables us to identify whether mental distress and RFs recover after the stressor [11], and (2) indicates which RFs may be promising treatment targets at times of stress. To this end, we designed the RESIST (Resilience Study) study, in which we assessed perceived stress, mental distress, and RFs in Cambridge University medical students before, during, and after their yearly exam period.

A recent meta-analysis based on 122,356 medical students from 43 countries showed that the prevalence rate for depressive symptoms was 27.2% (range of individual studies: 1.4%-73.5%) [12]. This prevalence rate was higher than that for population-representative peers of a similar age [12], suggesting that medical students are a high-risk population. In addition to depression, anxiety and general distress levels were also found to be elevated in students pursuing medical degrees, when compared with population-representative samples [13]. Exam stress has been identified as a potential trigger for mental ill-health in medical students [13-15]. Hence, research suggests that medical students are prone to the development of mental health problems, particularly during times of high and unavoidable exam stress.

The RESIST study is designed to capture (1) a period of moderate stress during the university term several months before exams, (2) a period of high stress during exam time, and (3) a period of what we expected to be low or moderate stress after exams (ie, during the summer vacation for many students). In addition to perceived stress and mental distress, we assessed 8 putative *individual-level* RFs (eg, self-esteem), 5 putative *family-level* RFs (eg, parental involvement), and 1 putative *community-level* RF (eg, friendship support; a complete list of assessed RFs is provided in the *Methods* section). Importantly, all of these RFs are derived from our preregistered systematic review and are thus empirically supported [3]. In our review, RFs were defined as those factors that moderate and/or mediate, and thereby mitigate, the detrimental relationship between adversity and subsequent mental distress [3]. Moreover, all assessed RFs are expected to be amenable to intervention, as only those can be successfully targeted by mental health services [3].

### Objectives

With the RESIST study, we intend to shed light on which RFs lend themselves best as prevention targets (before the stressor) and which lend themselves best as treatment targets (at times of stress) for the mitigation of mental health problems that are triggered or accelerated by a natural stressor. Therefore, the RESIST study may lay the foundations necessary to inform student support services, mental health services, as well as resilience and transdiagnostic mental health theory. Given that our design relies strongly on the assumption that stress and mental distress levels increase during the exam period, we here conduct proof-of-principle analyses to investigate whether this is indeed the case.

## Methods

### Design

**Figure 1:**
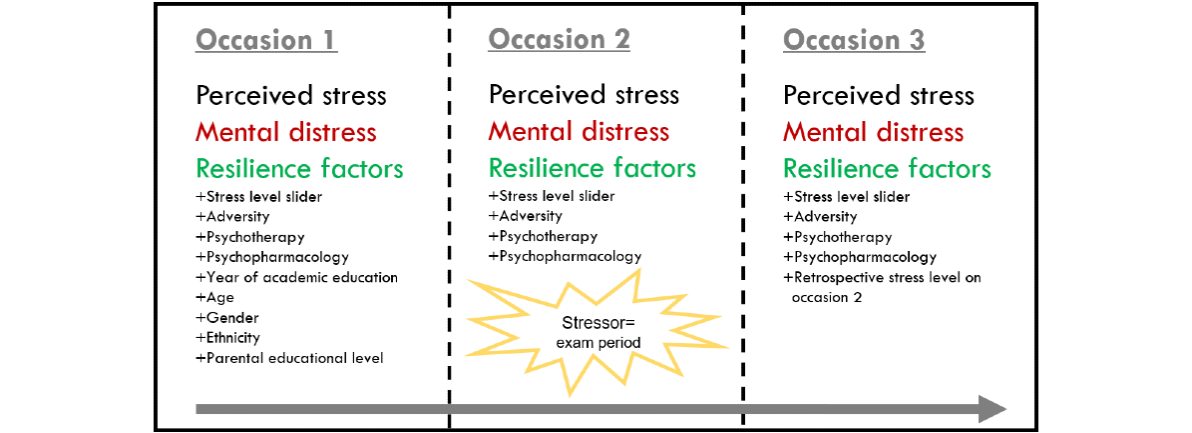
Study design. The Figure depicts the measures that have been assessed on the 3 occasions.

RESIST is a cohort study with 3 occasions and a within-subject (natural) stress manipulation (ie, the exam period). Occasion 1 took place in a nonexam period during the university term (February and March 2018). Occasion 2 took place during the end-of-year exam period (approximately April to June 2018, depending on the timing of the exam period). Occasion 3 took place after the exam period, at the end of the term for year 6 students (for whom exams are earlier; approximately end of May to mid-July 2018), and in the summer vacation or autumn for year 1-5 students (approximately mid-August to mid-October 2018; Figure 1). At all 3 occasions, students were asked to complete a survey containing a series of web-based questionnaires. At occasion 2, students were provided with the questionnaires 3 weeks before their first final exam. The questionnaires had to be completed before attempting the last final exam. In this way, all participants were exposed to the same type of naturally occurring external stressor. We assessed perceived stress, mental distress, RFs, therapeutic treatment, and psychopharmacology usage on all 3 occasions. Past adversity, year of academic education, age, gender, ethnicity, and parental educational level were assessed at occasion 1, and adversity occurring in between the occasions was assessed at occasions 2 and 3 (Figure 1).

### Sample

We recruited first- to sixth-year Cambridge medical students (from a cohort of approximately 1464 students). The inclusion criterion was that students had to be aged at least 18 years. Participants received monetary reimbursement for partaking (web-based vouchers: £5 [US $6.75] for occasion 1, £7 [US $9.50] for occasion 2, and £5 [US $6.75] for occasion 3). Participants who completed all 3 occasions were additionally enrolled in a prize draw (prize: five £50 [US $67.50] web-based vouchers). The maximum possible sample size we could have included was 800 participants, as we had a limited amount of money that we could spend on participant reimbursement. As a minimum sample size, we aimed for 225 participants. This is because we calculated that for a Gaussian regression-based model (minimum sample size = [((p × (p − 1))/2) × 5] [16-18]) with 9 RFs and mental distress (resulting in p=10 variables) we would need at least 225 participants ([((10 × (10 − 1))/2) × 5] = 225), given no longitudinal missingness (which was highly unlikely). Eventually, 451 participants took part on occasion 1 but some dropped out on the other 2 occasions (occasion 2: n=275; occasion 3: n=283; Table 1).

**Table 1:** Sample size overview (N=451).

Sample	Occasion		
	Occasion 1, n (%)	Occasion 2^a^, n (%)	Occasion 3^a^, n (%)
Taken part on, at least, 1 occasion	451 (100)	275 (61.0)	283 (62.8)
Taken part on, at least, 2 occasions	324 (71.8)	275 (61.0)	283 (62.8)
Taken part on occasion 2 but not 3	41 (9.1)	41 (9.1)	N/A^b^
Taken part on occasion 3 but not 2	49 (10.9)	N/A	49 (10.9)
Taken part on all occasions	234 (51.9)	234 (51.9)	234 (51.9)

^a^On occasions 2 and 3, only participants who had already taken part on occasion 1 were invited.

^b^N/A: not applicable.

### Procedure

The students received a web-based link to the questionnaire (survey software: REDCap [Research Electronic Data Capture]) via email. To prevent double partaking, we sent personalized emails with unique links to the students. We also advertised the study during lectures. Students who had not already participated received reminder emails until the end of the study occasion. For the first occasion, the link expired after 8 weeks. We sent the link for the second occasion approximately 3 weeks before the students’ first final exam and asked the students to confirm that they will submit the survey before their last final exam of the academic year. For year 6 students, the study link for the third occasion was sent out approximately a month before the end of the summer term. For year 1-5 students, the study link for the third occasion was sent 6 weeks before the start of the new academic year (the link for the third occasion expired after 8 weeks for all students).

### Measures

Before we finalized the web-based survey, we performed a user review with volunteering medical students. On the basis of this pilot study, we evaluated whether the survey was easily understandable and acceptable. We adapted small features, mainly regarding the survey layout (importantly, these data were not part of the study). On occasion 1, a total of 139 items were assessed.

#### Demographic and Clinical Characteristics

We assessed 8 demographic and clinical variables: academic course, year of academic education, gender, age, ethnicity, parental educational level, psychotherapeutic treatment, and psychopharmacology intake (ie, prescribed drugs).

#### Perceived Stress

We assessed the stress level during the last month using a 4-item short form of the validated Perceived Stress Scale (PSS) [19]. The self-report items assess topics such as confidence in handling problems and overcoming difficulties [19]. The short form of the PSS has been reported to have a Cronbach α of .72 [19]. In our sample, the PSS had an acceptable reliability (Cronbach α=.75; coefficient Ω=0.75). Moreover, we assessed the global stress severity during the last month on a zero-to-hundred slider.

#### Mental Distress

We assessed general mental health using the 12-item version of the General Health Questionnaire (GHQ-12) [20]. The GHQ-12 provides a broad indication of mental health and well-being across the spectrum from good-to-poor mental health but does not act as a measure of diagnosis for mental illness. The self-report items assess topics such as concentration, sleep, or happiness (measured on a 4-point scale). In a previous study, the GHQ was found to have a Cronbach α of between .78 and .95 [21]. The GHQ-12 previously had a mean area under the Receiver Operating Characteristic curves of 0.88 [22]. In our sample, the GHQ-12, in the remainder referred to as mental distress, had a good reliability (Cronbach α=.88; coefficient Ω=0.89).

#### RFs: Individual Level

We assessed 7 *individual-level* RFs that were empirically supported in our systematic review [3]: high distress tolerance, low ruminative reflection, low ruminative brooding, high self-esteem, high cognitive reappraisal, low expressive suppression, and low aggression potential. The content and psychometric details are shown in Table 2. In Table 2, we report Cronbach α values for previous studies, given that this was the reported internal consistency metric. However, for RESIST we report both Cronbach α and coefficient Ω [23], for completeness.

**Table 2:** Details of the resilience factor measures.

RFs^a^			Content and psychometric information
**Individual-level RFs**			
	High distress tolerance	6-item subscale of the DTS^b^ (15 items in total) [24] Self-report items assessing distress tolerance levels such acceptability of being upset Previous research found a good reliability (DTS Cronbach α=.82 to .85; 6-item tolerance subscale Cronbach α=.82 to .84) [24] In RESIST^c^, the distress tolerance subscale had a good reliability (Cronbach α: o1^d^=.82, o2^e^=.84, o3^f^=.83; coefficient Ω: o1=0.82, o2=0.85, o3=0.83)	
	Low ruminative reflection	5-item reflective rumination subscale of the RRS^g^ (22 items in total) [25] Self-report items assessing ruminative reflection levels such as trying to understand why you have a negative mood or why you feel in a given way Previous research found an acceptable reliability (RRS Cronbach α=.90; 5-item reflective rumination subscale Cronbach α=.72) [25] In RESIST, the reflective rumination subscale had an acceptable reliability (Cronbach α: o1=.75, o2=.76, o3=.79; coefficient Ω: o1=0.76, o2=0.76, o3=0.80)	
	Low ruminative brooding	5 item brooding subscale of the RRS (22 items in total) [25] Self-report items assessing brooding levels such as why things do not work out better or why other people do not have comparable problems Previous research found an acceptable reliability (RRS Cronbach α=.90; 5-item brooding subscale Cronbach α=.77) [25] In RESIST, the brooding subscale had an acceptable reliability (Cronbach α: o1=.75, o2=.79, o3=.77; coefficient Ω: o1=0.76, o2=0.80, o3=0.78)	
	High self-esteem	10 items of the RSES^h^ [26] Self-report items assessing positive self-esteem levels such as being capable of doing things well and negative self-esteem levels such as feeling useless Previous research found a good reliability (RSES Cronbach α=.88) [26,27] In RESIST, the RSES had an excellent reliability (Cronbach α: o1=.93, o2=.94, o3=.92; coefficient Ω: o1=0.93, o2=0.94, o3=0.92)	
	High cognitive reappraisal	6-item cognitive reappraisal subscale of the ERQ^i^ (10 items in total) [28,29] Self-report items assessing cognitive reappraisal levels such as changing the content of thoughts to achieve a less negative or more positive mood Previous research found an acceptable reliability (6-item cognitive reappraisal subscale Cronbach α=.79) [28,29] In RESIST, the cognitive reappraisal subscale had a good reliability (Cronbach α: o1=.83, o2=.87, o3=.88; coefficient Ω: o1=0.83, o2=0.87, o3=0.88)	
	Low expressive suppression	4-item expressive suppression subscale of the ERQ (10 items in total) [28,29] Self-report items assessing expressive suppression levels, that is, the extent to which individuals suppress positive and negative emotions Previous research found an acceptable reliability (4-item expressive suppression subscale Cronbach α=.73) [28,29] In RESIST, the expressive suppression subscale had an acceptable reliability (Cronbach α: o1=.75, o2=.73, o3=.76; coefficient Ω: o1=0.78, o2=0.76, o3=0.78)	
	Low aggression potential	12-item BAQ^j^ [30] Self-report about aggression levels including physical aggression, verbal aggression, anger, and hostility Previous research found an acceptable-to-good reliability (BAQ Cronbach α=.76-.83) [30] In RESIST, the BAQ had an acceptable reliability (Cronbach α: o1=.78, o2=.80, o3=.79; coefficient Ω: o1=0.79, o2=0.81, o3=0.78)	
**Family-level RFs**			
	High immediate family support	6-item abbreviated version of the PSS-Fa^k^ (20 items in total) [31,32] Self-report about family support, such as getting emotional support and having someone who can help out solving problems Previous research found a low reliability (PSS-Fa Cronbach α=.90; 6-item abbreviated PSS-Fa Cronbach α=.69) [31,32] In RESIST, the abbreviated PSS-Fa had a good reliability (Cronbach α: o1=.88, o2=.83, o3=.85; coefficient Ω: o1=0.88, o2=0.83, o3=0.85)	
	High extended family support	13-item KSS^l^ [33,34] Self-report about extended family and kinship support, such as asking relatives for advice when making decisions or confiding in relatives when having a problem Previous research found an acceptable-to-good reliability (KSS Cronbach α=.72-.89) [33,34] In RESIST, the KSS had an excellent reliability (Cronbach α: o1=.92, o2=.91, o3=.93; coefficient Ω: o1=0.92, o2=0.91, o3=0.93)	
	High family cohesion	5-item family cohesion subscale of the SFI-II^m^ (36 items in total) [35,36] Self-report about family cohesion, such as preferably spending time with the family rather than with others Previous research found a low reliability (SFI-II Cronbach α=.91; 5-item family cohesion subscale Cronbach α=.60) [36] In RESIST, the family cohesion subscale had a good reliability (Cronbach α: o1=.86, o2=.84, o3=.87; coefficient Ω: o1=0.87, o2=0.85, o3=0.88)	
	High positive parenting	6-item positive parenting subscale of the APQ^n^ (42 items in total) [37,38] Child (ie, in our study, young adult) report about positive parenting, such as positive encouragement, compliments, and praise from parents for doing a good job (ie, for the time when the participants lived with their parents) [39] Previous research found an acceptable reliability (6-item positive parenting subscale Cronbach α=.72-.75) [37,38] In RESIST, the positive parenting subscale had a good reliability (Cronbach α: o1=.87, o2=.88, o3=.88; coefficient Ω: o1=0.87, o2=0.88, o3=0.88)	
	High parental involvement	10-item parental involvement subscale of the APQ (42 items in total) [37,38] Child (ie, in our study, young adult) report about parental involvement levels, such as doing activities together and asking about the child’s friends and school performances (ie, for the time when the participants lived with their parents) [39] We collapsed separate statements for “moms” and “dads” into a single “parent” statement (eg, original: “Your mom talks to you about your friends. How about your dad?,” adaptation: “Your parents talk to you about your friends.” as done in previous studies, such as in van Harmelen et al [39]) Previous research found an acceptable-to-good reliability (10-item parental involvement subscale Cronbach α=.71-.83) [37,38] In RESIST, the parental involvement subscale had a good reliability (Cronbach α: o1=.87, o2=.89, o3=.87; coefficient Ω: o1=0.87, o2=0.90, o3=0.87)	
**Community-level RF**			
	High friendship support	6-item abbreviated version of the PSS-Fr^o^ (20 items in total) [31,32] Self-report about friendship support, such as getting moral support and having companionship Previous research found an acceptable reliability (PSS-Fr Cronbach α=.88; 6-item abbreviated PSS-Fr Cronbach α=.75) [31,32] In RESIST, the abbreviated PSS-Fr had a good reliability (Cronbach α: o1=.80, o2=.81, o3=.79; coefficient Ω: o1=0.80, o2=0.81, o3=0.79)	

^a^RF: resilience factor.

^b^DTS: Distress Tolerance Scale.

^c^RESIST: Resilience Study.

^d^o1: occasion 1.

^e^o2: occasion 2.

^f^o3: occasion 3.

^g^RRS: Ruminative Response Scale.

^h^RSES: Rosenberg Self-Esteem Scale.

^i^ERQ: Emotion Regulation Questionnaire.

^j^BAQ: Brief Aggression Questionnaire.

^k^PSS-Fa: Perceived Social Support from Family Scale.

^l^KSS: Kinship Social Support Measure.

^m^SFI-II: Self-Report Family Inventory Version II.

^n^APQ: Alabama Parenting Questionnaire.

^o^PSS-Fr: Perceived Social Support from Friends Scale.

#### RFs: Family Level

We assessed 5 *family-level* RFs that were empirically supported in our systematic review [3]: high immediate family support, high extended family support, high family cohesion, high positive parenting, and high parental involvement. The content and psychometric details are shown in Table 2. As items of the family-related scales may be hard to answer for participants if they have spent a large amount of their childhood in care homes or frequently changed foster families, we added some specific instructions to those survey parts (Supplement I in Multimedia Appendix 1).

#### RFs: Community Level

We assessed 1 *community*-level resilience factor, which was empirically supported in our systematic review [3]: high social support, here specifically friendship support. The content and psychometric details are shown in Table 2.

#### Adversity

Environmental childhood and youth adversity was assessed using an updated version of the 12-item Youth Trauma Scale (YTS) [40]. The 12 self-report items assess topics such as sexual abuse or severe mental or physical illnesses within the family. A complete list of items and assessment details can be found in Supplement II in Multimedia Appendix 1. The original scale was found to have a low internal consistency (Cronbach α=.63-.67). We adapted the questionnaire [40] so that the words *parent and sibling* were supplemented with *significant other*, as our participants were at an age at which some might have invested in significant interpersonal relationships outside the family, such as long-term romantic partners. Besides the presence versus absence of adversity, the questionnaire also assessed the severity of questions for which the presence of the respective adversity was positively confirmed. Moreover, for positively confirmed questions, we further assessed the frequency or duration of the adversity as well as during which age bins the adversity had taken place. We did not assess the frequency or duration for two of the adversity items: (1) “Were you separated from one of your parents for more than 1 year?” and (2) “Was either of your parents unemployed for more than 1 year when they wanted to be working?” as those have an inherent time requirement of at least one year. To also assess the potential criminality of parents, siblings, or significant others, we added such an item to the original scale (“Did parents, siblings, or significant others engage in criminal activities severe enough to cause significant stress or worry?”). In summary, we assessed the presence versus absence of the 13 adversities, and if present, the severity, frequency, and the age bin in which the respective adversity experience occurred. Owing to the adaptations we made, we will thoroughly evaluate the psychometrics of the amended scale in a separate manuscript [40].

Furthermore, we assessed the psychological maltreatment and neglect subscales of the Comprehensive Child Maltreatment Scale (CCMS) [41]. The psychological maltreatment subscale consists of 3 items that assess topics such as how frequently the individual was yelled at, ridiculed, or provoked [41]. The neglect subscale consists of 3 items that assess topics such as whether the individual was provided with sufficient warmth from family members, sufficient nutrition, and protection [41]. In a previous study, the CCMS psychological maltreatment subscale was found to have a Cronbach α of .78, and the CCMS neglect subscale had a Cronbach α of .84 [41]. The only adaptation we made to this scale was that we did not assess the questions up to the age of 13 years [41] but up to the age of 18 years. In our sample, the CCMS psychological maltreatment (Cronbach α=.81; coefficient Ω=0.81) and the CCMS neglect subscale both had a good reliability (Cronbach α=.84; coefficient Ω=0.81).

### Measures for Occasion 2

We assessed psychotherapeutic treatment and the use of psychopharmacological drugs for the period between occasions 1 and 2.

We assessed perceived stress (Cronbach α=.74; coefficient Ω=0.75) and global stress severity in the same way as described for occasion 1, while this time specifically focusing on the exam period. Moreover, we quantified the number of exams (completed and not yet completed). The mental distress (Cronbach α=.89; coefficient Ω=0.89) and RF levels (for reliability coefficients, see Table 2) were assessed in the same way as on occasion 1.

Adversity was again assessed with the updated version of the YTS [40]; however, this time we only asked for experiences during the period between occasions 1 and 2 (“This section will ask you about your experiences of potentially traumatic events. Please indicate whether you have experienced those since the last time you filled in this questionnaire*.*”). Importantly, all the YTS adaptations explained above were applied again. We did not reassess the CCMS subscales [41], as we used the psychological maltreatment and neglect subscales to measure maltreatment during childhood and the teenage years, while living at home. Therefore, the subscales were not suitable for the time between occasions 1 and 2.

A total of 127 items were assessed.

### Measures for Occasion 3

We assessed psychotherapeutic treatment and the use of psychopharmacological drugs for the period between occasions 2 and 3.

We assessed perceived stress (Cronbach α=.74; coefficient Ω=0.75) and global stress severity in the same way as described for occasion 1. Moreover, we asked the students whether they had stressful or significant work during the last 4 weeks. Mental distress (Cronbach α=.90; coefficient Ω=0.91) and RF levels (for reliability coefficients, see Table 2) were assessed in the same way as on occasions 1 and 2.

Adversity was again assessed with the updated version of the YTS [40], which this time asked for experiences during the period between occasions 2 and 3. All YTS adaptations explained for occasions 1 and 2 were applied again. We did not reassess the CCMS subscales [41] for the same reason as on occasion 2.

As occasion 2 took place during an exam period, we assumed that it could potentially be the case that missingness may not be completely random but dependent on the students’ stress level during occasion 2. Therefore, we asked all participants how stressful the exam period had been.

A total of 129 items were assessed.

### Ethical Considerations

#### Informed Consent and Safety Considerations

Before starting the content part of the web-based survey, participants were asked to read the information sheet, which contained the major study aims, and to complete a consent form. Before completion of the survey, participants were enabled to download their consent form and the information sheet. Moreover, we provided details on how to get help and support, in case the study would bring up difficult feelings or in case a participant would want to report childhood maltreatment or a crime, in a mental health services information sheet, which could be downloaded from the web-based survey. Further details regarding participant safety considerations are provided in Supplement III in Multimedia Appendix 1.

#### Ethics Approval and Funding Information

RESIST was approved by the Cambridge Psychology Research Ethics Committee (PRE.2017.096). RESIST was funded by JF’s Medical Research Council Doctoral Training Grant and by POW’s personal research account.

### Analytic Methods for the Proof-of-Principle Analyses

#### Handling Missing Data

To include both participants with incomplete and complete data, we used a full information maximum likelihood (FIML) estimator. The use of this estimator has been shown to function well in longitudinal structural equation models. For example, Kievit et al [42] report that “FIML usually performs as well or better than alternative methods.” We decided to treat missing data, instead of performing a complete case analysis, to increase statistical power, reduce standard errors, decrease the probability of biased parameter estimates, and improve generalizability. We tested several potential variables as predictors for missing data patterns, including (1) perceived stress, global stress severity (ie, stress slider), mental distress, psychotherapeutic treatment, and psychopharmacological treatment on all 3 occasions; (2) gender, ethnicity, academic year, age, and childhood adversity on occasion 1; and (3) retrospective subjective stress levels for occasion 2 assessed on occasion 3. This was primarily done to enhance the understanding of missingness in the RESIST sample, and because variables that qualify as predictors for missing data patterns can, in conjunction with an FIML estimator, be used as auxiliary variables and thereby potentially enhance the estimation precision.

#### Latent Growth Models

We conducted a series of latent growth models (LGMs) to explore the mean change trajectory of perceived stress and mental distress over the 3 occasions. We fixed the slope loading of occasion 2 to 1, expecting this occasion to have the highest level of perceived stress (and mental distress), and the slope loading of occasion 3 to 0, expecting this occasion to have a lower level of perceived stress (and mental distress) than occasion 2. Hence, the slope loading of occasion 1 was freely estimated and provides an indication of where the (scaled) mean level lies in comparison to occasion 2 (fixed to 1) and occasion 3 (fixed to 0). We conducted the LGMs with invariant residual variances for the 3 occasions (M1). To test whether our latent growth model is significantly different from a *no-change* trajectory (ie, no change in overall mean levels), we estimated an additional LGM (M2) with the latent slope mean set to 0, modeling no overall change. In sum, we mainly used the models to identify the change trajectories of perceived stress and mental distress and to test whether these trajectories fit better than a *no-change* trajectory. For completeness, we refitted the LGMs with freely estimated residual variances (Supplement IV in Multimedia Appendix 1).

#### Bivariate Latent Change Score Models

We conducted a series of bivariate latent change score models (BLCSMs; as described by Kievit et al [42]) to investigate the change of perceived stress and mental distress in conjunction. More specifically, we conducted 3 BLCSMs, 1 for each pairwise combination of the 3 occasions, to allow for direct comparisons without estimating overly complex models. To enable the computation of the models, we used the standard BLCSM estimation (additional details are provided by Kievit et al [42]). We then investigated the relationship between perceived stress and mental distress on the earlier occasion as well as the relationship between the change scores of perceived stress and mental distress on the later occasion. Moreover, we investigated the autoregressive paths of perceived stress and mental distress with their respective change scores as well as the potentially mutualistic relationship between perceived stress and mental distress, that is, perceived stress predicting change in mental distress and mental distress predicting change in perceived stress.

#### Data and Analysis-Code Availability

All analyses were conducted in R version 3.5.1 (The R Foundation) [43], mainly using the packages lavaan [44] and semTools [45]. The analysis script can be found on the Open Science Framework [46] and the anonymized data used for the analyses in this manuscript have been uploaded to the Cambridge Data Repository [47].

## Results

### Demographic and Clinical Characteristics

#### Sample

Students were approximately uniformly distributed over all 6 academic years, with percentages ranging from 12.4% (56/451) to 20.6% (93/451) per year. A total of 57.4% (259/451) of the students were female (1.3% [6/451] preferred not to answer) and 58.3% (263/451) were White. Most students were between 18 and 23 years of age and had parents with higher education after secondary school. About 13.5% (61/451) of the students received psychotherapeutic treatment and 10.9% (49/451) received psychopharmaceutic treatment in the 6 months before occasion 1. Table 3 contains the descriptive statistics for all students who took part on occasion 1. Supplement V in Multimedia Appendix 1 contains the same table, with the inclusion of all students who provided data for at least two occasions (n=324).

**Table 3:** Demographic and clinical characteristics for the overall sample (N=451).

Characteristics				Sample size per answer category, n (%)
**Academic year**				
	First year		93 (20.6)	
	Second year		83 (18.4)	
	Third year		66 (14.6)	
	Fourth year		88 (19.5)	
	Fifth year		56 (12.4)	
	Sixth year		65 (14.4)	
**Gender^a^**				
	Female		259 (57.4)	
	Male		185 (41.0)	
	Prefer not to say		6 (1.3)	
**Age^b^ (years)**				
	18-20		170 (37.7)	
	21-23		196 (43.5)	
	24-26		65 (14.4)	
	≥27		17 (3.8)	
**Ethnicity^c^**				
	White		263 (58.3)	
	Non-White		184 (40.8)	
**Therapeutic treatment (in the 6 months before occasion 1)**				
	No		390 (86.5)	
	Yes		61 (13.5)	
**Psychopharmaceutic treatment (in the 6 months before occasion 1)**				
	No		402 (89.1)	
	Yes		49 (10.9)	
**Education (further or higher education after secondary school)**				
	**Mother**			
		Yes	359 (79.6)	
		No	88 (19.5)	
		Unknown	4 (0.9)	
	**Father^a^**			
		Yes	369 (81.8)	
		No	77 (17.1)	
		Unknown	4 (0.9)	

^a^One student did not answer this question. Due to missingness, some percentages may not add up.

^b^Three students did not answer this question. Due to missingness, some percentages may not add up.

^c^Four students did not answer this question. Due to missingness, some percentages may not add up.

#### Perceived Stress and Mental Distress Variables

The mean levels suggest that perceived stress and mental distress increased from the time before the exams to the exam period and decreased after the exams to a lower level than before the exams. Table 4 depicts perceived stress and mental distress levels on the 3 occasions for participants who took part in the respective occasion. The corresponding table with only those participants who provided data for at least two occasions can be found in Supplement V in Multimedia Appendix 1.

**Table 4:** Perceived stress and mental distress levels for the 3 occasions.

Measure	Occasion 1		Occasion 2			Occasion 3	
	Score, mean (SD)	Participants, n	Score, mean (SD)	Participants, n	Score, mean (SD)		Participants, n
PSS^a^	10.42 (2.77)	451	11.61 (2.77)	274	9.89 (2.67)		282
GHQ-12^b^	25.40 (5.82)	445	27.39 (6.09)	273	23.31 (5.93)		282

^a^PSS: Perceived Stress Scale.

^b^GHQ-12: General Health Questionnaire, 12-item version.

### Missingness Predictors

Gender and ethnicity as well as psychopharmacological medication and global stress severity (ie, stress slider) on occasion 1 were identified as predictors for missing data patterns (see test results in Supplement VI in Multimedia Appendix 1). Therefore, we decided to include these as auxiliary variables in our analyses. However, as adding the auxiliary variables to our models did not result in positive definite residual matrices, we here describe the models without auxiliary variables. We provide the results with auxiliary variables in Supplement VI in Multimedia Appendix 1. Importantly, all results remained largely the same when auxiliary variables were added to the models. Moreover, as additional robustness analysis, we reconducted all main analyses, while this time excluding one potentially influential case. Once again, all results remained largely the same (Supplement VII in Multimedia Appendix 1).

### Latent Growth Models

#### Perceived Stress

**Figure 2:**
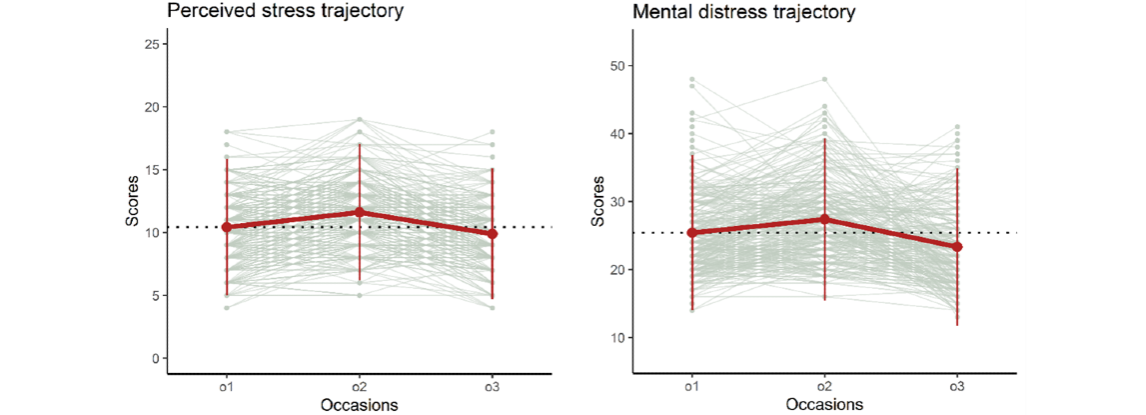
The left panel depicts the perceived stress (sum score mean level) trajectory and the right panel depicts the mental distress (sum score mean level) trajectory. The faded gray lines indicate person-level trajectories. The red line indicates the group-level sum score trajectory, which was averaged across the students. The dotted black line represents the group-level sum score for occasion 1. This was done solely to enhance the comparison with the other occasions. o1: occasion 1; o2: occasion 2; o3: occasion 3.

The LGM showed that, on average, students experience most perceived stress during exams (occasion 2: slope loading fixed to 1) and least perceived stress after the exams (occasion 3: slope loading fixed to 0); before the exams, they experienced more perceived stress than after the exams, but less than during the exams (occasion 1: estimated slope loading=0.29; Table 5). The mean level trajectory is shown in Figure 2 (left panel). We further found that the model estimating the change trajectory of perceived stress fits significantly better than the *no-change* model (*χ*^2^_1_=72.4; *P*<.001; fit indices are presented in Table 6), indicating that perceived stress changed significantly over the 3 occasions. Additional post-hoc analyses confirmed that the mean levels differed significantly between all three occasions.

**Table 5:** Latent growth model summary.

Model			Slope loading, occasion 1		Slope loading, occasion 2		Slope loading, occasion 3		Intercept mean		Slope mean		Residual variance, occasion 1		Residual variance, occasion 2		Residual variance, occasion 3		Intercept slope covariance
**Perceived stress**																			
	M1^a^	0.29		1.00		0		9.92		1.79		3.78		3.78		3.78		0.45	
	M2^b^	0.52		1.00		0		10.54		0		3.68		3.68		3.68		−1.35	
	M1^c^	0.29		1.00		0		9.91		1.79		3.75		3.75		3.75		0.41	
**Mental distress**																			
	M1	0.60		1.00		0		23.21		4.08		16.85		16.85		16.85		−8.71	
	M2	−4.41		1.00		0		25.44		0		37.50		37.50		37.50		−1.65	
	M2^c^	0.67		1.00		0		25.26		0		16.74		16.74		16.74		−18.07	

^a^M1: freely estimated trajectory model.

^b^M2: no-change trajectory model.

^c^Variance for the latent slope constrained to >0 to render it nonnegative.

**Table 6:** Latent growth model fit.

Model		AIC^a^	BIC^b^	CFI^c^	TLI^d^	RMSEA^e^	SRMR^f^	Chi-square (*df*)	BICw^g^ (%)	AICw^h^ (%)
**Perceived stress**										
	M1^i^	4718.05	4746.83	0.98	0.96	0.07	0.04	6.5 (2)	100	100
	M2^j^	4811.92	4836.59	0.47	0.47	0.27	0.18	102.3 (3)	0	0
	M1^k^	4718.06	4746.84	0.98	0.96	0.07	0.04	6.5 (2)	100	100
**Mental distress**										
	M1	6295.92	6324.66	0.94	0.91	0.09	0.05	9.2 (2)	100	100
	M2	6366.44	6391.08	0.32	0.32	0.24	0.19	81.7 (3)	0	0
	M2^k^	6364.52	6389.17	0.34	0.34	0.24	0.18	79.8 (3)	0	0

^a^AIC: Akaike information criterion.

^b^BIC: Bayesian information criterion.

^c^CFI: confirmatory fit index.

^d^TLI: Tucker-Lewis fit index.

^e^RMSEA: root mean square error of approximation.

^f^SRMR: standardized root mean square residual.

^g^BICw%: weight percentage for the Bayesian information criterion (compared to the respective other model); the higher the weight, the more in favor is the model.

^h^AICw%: weight percentage for the Akaike information criterion (compared to the respective other model); the higher the weight, the more in favor is the model.

^i^M1: freely estimated trajectory model.

^j^M2: no-change trajectory model.

^k^Variance for the latent slope constrained to >0 to render it nonnegative.

#### Mental Distress

The LGM showed that, on average, students experience most mental distress during exams (occasion 2, slope loading fixed to 1) and least mental distress after exams (occasion 3, slope loading fixed to 0); before the exams, they experienced more mental distress than after the exams, but less than during the exams (occasion 1, estimated slope loading=0.60; Table 5). The mean level trajectory is shown in Figure 2 (right panel). We further found that the model estimating the change trajectory of mental distress fits significantly better than the *no-change* model (*χ*^2^_1_=58.8; *P*<.001; fit indices are presented in Table 6), indicating that mental distress changed significantly over the 3 occasions. Additional post-hoc analyses confirmed that the mean levels differed significantly between all three occasions.

### Bivariate Latent Change Score Models

#### Occasions 1-2

**Figure 3:**
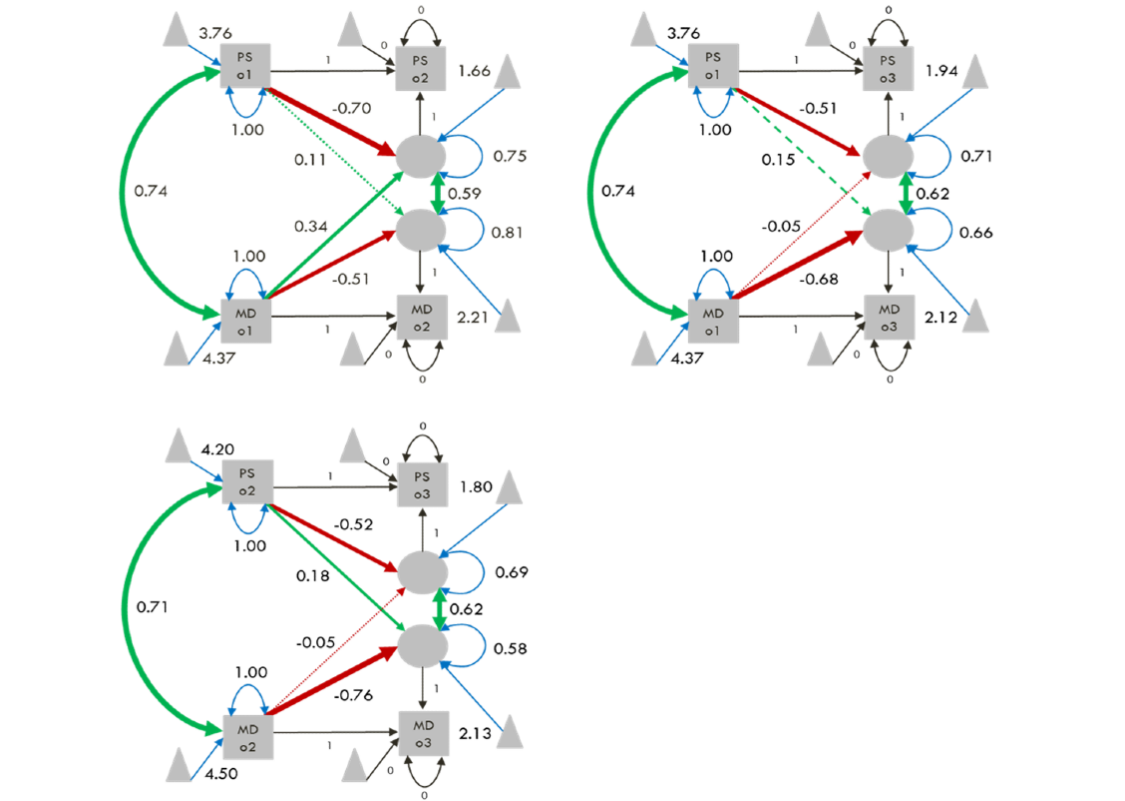
Bivariate Latent Change Score Models. The upper-left panel depicts occasions 1-2, the upper-right panel depicts occasions 1-3, and the lower-left panel depicts occasions 2-3. Green arrows represent positive associations, red arrows represent negative associations, black arrows represent fixed parameters, and blue arrows represent estimated intercepts and variances. Double-headed arrows represent covariances and variances, and single-headed arrows represent intercepts, regressions, and autoregressions. Solid lines indicate a significant association (*P*<.05), dashed lines indicate marginal association (.05≥*P*<.10), and dotted lines indicate nonsignificant association (*P*≥.10). Gray squares represent manifest variables, gray circles represent latent variables, and gray triangles represent intercepts. All depicted estimates are standardized. MD: mental distress; o1: occasion 1; o2: occasion 2; o3: occasion 3; PS: perceived stress.

The BLCSM showed that perceived stress and mental distress on occasion 1 are significantly positively associated, which indicates that students with higher perceived stress report on average higher mental distress (Figure 3, upper-left panel; for exact coefficients, see Supplement VIII in Multimedia Appendix 1). Similarly, changes in perceived stress and mental distress from occasion 1 to 2 are significantly positively associated, which indicates that students with more increase in perceived stress report on average also a greater increase in mental distress. The BLCSM revealed a significant and negative autoregressive effect for perceived stress and its change score, which indicates that higher perceived stress on occasion 1 results, on average, in less increase in perceived stress from occasion 1 to 2. Equally, mental distress had a significant and negative autoregressive effect with its change score, indicating that higher mental distress on occasion 1 results, on average, in less increase in mental distress from occasion 1 to 2. Perceived stress was not significantly associated with the change in mental distress from occasion 1 to 2. However, mental distress was significantly positively associated with the change in perceived stress from occasion 1 to 2, which indicates that higher mental distress on occasion 1 results on average a greater increase in perceived stress from occasion 1 to 2.

#### Occasions 1-3

The BLCSM showed that changes in perceived stress and mental distress from occasion 1 to 3 are significantly positively associated, which indicates that individuals with a greater decrease in perceived stress report, on average, also a greater decrease in mental distress (Figure 3, upper-right panel; for exact coefficients, see Supplement VIII in Multimedia Appendix 1). Moreover, the BLCSM revealed a significant and negative autoregressive effect for perceived stress and its change score, which indicates that higher perceived stress on occasion 1 results, on average, in a greater decrease in perceived stress from occasion 1 to 3. Equally, mental distress had a significant and negative autoregressive effect with its change score, indicating that higher mental distress on occasion 1 results, on average, in a greater decrease in mental distress from occasion 1 to 3. Mental distress was not significantly associated with change in perceived stress from occasion 1 to 3. Perceived stress was not significantly associated with change in mental distress from occasion 1 to 3; however, the P value was marginal (β=.15; *P*=.06). If one would opt to interpret the directionality of the effect, it would suggest that, on average, higher perceived stress is marginally associated with less decrease in mental distress from occasion 1 to 3. However, given that the effect was not significant, we suggest to err on the side of caution and shall not interpret it.

#### Occasions 2-3

The BLCSM showed that perceived stress and mental distress on occasion 2 are significantly positively associated, which indicates that individuals with higher perceived stress report on average higher mental distress (Figure 3, lower-left panel; for exact coefficients, see Supplement VIII in Multimedia Appendix 1). Similarly, changes in perceived stress and mental distress from occasion 2 to 3 are significantly positively associated, which indicates that individuals with a greater decrease in perceived stress report, on average, also a greater decrease in mental distress. Moreover, the BLCSM revealed a significant and negative autoregressive effect for perceived stress and its change score, which indicates that higher perceived stress on occasion 2 results on average in a greater decrease in perceived stress from occasion 2 to 3. Equally, mental distress had a significant and negative autoregressive effect with its change score, indicating that higher mental distress on occasion 2 results on average in a greater decrease in mental distress from occasion 2 to 3. Mental distress was not significantly associated with change in perceived stress from occasion 2 to 3. However, perceived stress was significantly positively associated with change in mental distress from occasion 2 to 3, which indicates that higher perceived stress results on average in less decrease in mental distress from occasion 2 to 3.

## Discussion

### Conclusions: Proof-of-Principle Analyses

Both perceived stress and mental distress were lower before the exams (ie, during the regular university term) than during the exam period, but higher before the exams than after the exams. Higher mental distress during term time was, on average, associated with a greater increase in perceived stress from the term time to the exam period, when controlling for perceived stress levels during the term time. Hence, students who already had mental health problems before the exam period were most prone to develop increased levels of stress during the exam period. Higher perceived stress during the exam period was, on average, associated with less recovery of mental distress after the exam time, when controlling for mental distress levels during exams. Thus, students who reported high stress during the exam period were less successful (or quick) in recovering from mental distress. Overall, we found that higher mental health problems before the exams increase the risk of developing more perceived stress during the exams, and higher perceived stress during the exams in turn increases the risk of a less successful (or quick) recovery of mental distress after exams.

### Future Research and Outcomes

#### Plans for Research Questions and Analyses

Future analyses on the RESIST data are primarily set out to shed light on which RFs lend themselves best as prevention targets (before the stressor) and which as treatment targets (at times of stress) for the mitigation of mental health problems that are triggered or accelerated by natural exam stress. Therefore, the RESIST study may lay the foundations necessary to inform student support services, as well as mental health services, as well as resilience and transdiagnostic mental health theory.

#### Plans for Outcome Dissemination and Data Availability

We aim to publish all articles that are based on RESIST data in peer-reviewed journals, ideally under an open access agreement. Alongside the manuscripts, we aim to release the related and anonymized data on the Cambridge Data Repository.

## Appendix

Multimedia Appendix 1 Contains 8 supplements (ie, Supplement I: Additional information for family-related questionnaires; Supplement II: Items of the adapted version of the Youth Trauma Scale; Supplement III: Participant safety considerations; Supplement IV: Latent Growth Models with varying residual variances; Supplement V: Demographic and clinical characteristics for the sample with data for at least 2 occasions; Supplement VI: Missingness predictors and analyses results when including the auxiliary variables; Supplement VII: Analyses results when excluding one potentially influential case; Supplement VIII: Model summaries and exact coefficients of the Bivariate Latent Change Score Models).

## Abbreviations

BLCSM: bivariate latent change score model
CCMS: Comprehensive Child Maltreatment Scale
FIML: full information maximum likelihood
GHQ: General Health Questionnaire
LGM: latent growth model
PSS: Perceived Stress Scale
REDCap: Research Electronic Data Capture
RESIST: Resilience Study
RF: resilience factor
YTS: Youth Trauma Scale
