# Supplementary material for: Tracking Stress, Mental Health, and Resilience Factors in Medical Students Before, During, and After a Stress-Inducing Exam Period: Protocol and Proof-of-Principle Analyses for the RESIST Cohort Study
Source: JMIR Form Res. 2021 Jun 8;5(6):e20128. doi: 10.2196/20128 (PMC8262546; doi:10.2196/20128)
Supplement: Multimedia Appendix 1 [file formative_v5i6e20128_app1.pdf]

TRACKING STRESS, MENTAL HEALTH AND RESILIENCE FACTORS IN MEDICAL STUDENTS, BEFORE, DURING AND AFTER A STRESS-INDUCING EXAM PERIOD

Protocol and Proof of Principle Analyses for the RESIST Cohort-Study

J. Fritz,[1,*] J. Stochl,[1,2] R.A. Kievit,[3,4] A.-L. van Harmelen,[1,3,5,A] & P.O. Wilkinson[1,A]

* Correspondence: Jessica Fritz, jf585@cam.ac.uk
[1] Department of Psychiatry, University of Cambridge, United Kingdom
[2] Department of Kinanthropology, Charles University, Czech Republic
[3] MRC Cognition and Brain Sciences Unit, University of Cambridge, United Kingdom
[4] Radboud University Medical Center, Donders Institute for Brain, Cognition, and Behavior, Nijmegen, the Netherlands
[5] Education and Child Studies, Leiden University, the Netherlands
[A] shared last author

Supplement I: Additional information for family-related questionnaires

As items of the family-related scales may be hard to answer for participants if they have spent a large amount of their childhood in care homes or frequently changed foster families, we added some specific instruction to those surveys parts, we

1. added an introductory text to the family-related questionnaires ('In case you haven't been growing up within 'traditional' family circumstances, please answer the questions for the average caregiving circumstances you have been growing up in.')
2. extend the introductory text for the four family-related questionnaires to explain the words 'family/immediate family', 'family members/members of my family', 'extended family', 'parents', and 'relatives' (see Table 1)
3. changed the word 'parents' to 'parents/carers'
4. made it clearer that participants can opt out of these questions, by asking two additional items for the first family-related questionnaire (see Table 2).

Table 1

Extended part of the introductory text of each of the four family-related questionnaires

**Questionnaire**	**Additional instructions**
Family cohesion questionnaire:	Please understand the word 'family' as immediate familial network irrespective of biological or non-biological relationships. Accordingly 'family members' are all members of your immediate familial network irrespective of having a biological or non-biological relationship with you.
Immediate family support questionnaire:	Please understand the word 'family' as immediate familial network irrespective of biological or non-biological relationships. Accordingly a 'member of your family' is a member of your immediate familial network irrespective of having a biological or non-biological relationship with you.
Extended family support questionnaire:	Please understand the word 'family' as immediate familial network irrespective of biological or non-biological relationships. Accordingly, please understand the word 'relatives' as extended familial network irrespective of biological or non-biological relationships.
Positive parenting and parental involvement questionnaire:	Please understand the word 'family' as immediate familial network irrespective of biological or non-biological relationships. Accordingly, please understand the word 'parents/carers' as caregivers irrespective of having a biological or non-biological relationship with you.

Table 2

Two additional items about the participants' individual family background.

**Item**	**Text**
Item 1	The first item asked the participants to indicate whether they grew up in a 'traditional family environment': '*I have been growing up within 'traditional family circumstances', which means that I have been growing up with at least one caregiver (irrespective of biological or non-biological caregiver) for most of my life up to the age of 18.*' (answer categories: '*yes*' or '*no*'). If participants answered this item with '*no*' an explanation box popped up and reminded the participants about the fact that they did not have to answer items if they preferred not to or if the items seemed inappropriate ('*Please remember, you do not have to answer the items if you prefer not to, or if the items seem inappropriate.*').
Item 2	The second item was conditional on the first item. Only if participants answered the first item with '*no*' they were asked whether they wanted to describe their individual family environment (i.e. in a text box with the instruction: '*If you want to, you can share the family environment you have been growing up in with us*'.

Supplement II: Items of the adapted version of the Youth Trauma Scale

Youth Trauma Scale (YTS; e.g. Schlechter, Fritz, & Wilkinson, 2019).

1. A death of a very close friend or family member?
    a. Please indicate whether you have experienced those at any time in your life.
        - yes
        - no
    b. In case of multiple experiences, please choose the most traumatic one for the following questions. How traumatic was this?
        - 1 - not at all traumatic
        - 4 - somewhat traumatic
        - 7 - extremely traumatic
    c. What was the frequency/duration of the experience?
        - once
        - twice
        - a week
        - a month
        - half a year
        - a year
        - more than a year
    d. How old were you when this experience took place/ started to take place?
        - birth to age 12
        - 12 to age 18
        - age 18+
2. A major upheaval between your parents (such as divorce, separation)?
3. A traumatic sexual experience (raped, molested, etc.)?
4. Were you the victim of violence (child abuse, mugged or assaulted other than sexual)?
5. Did you ever see a parent, sibling or significant other being the victim of violence?
6. Were you extremely ill or injured?
7. Was a parent, sibling, or significant other seriously ill or injured?
8. Did a parent, sibling, or significant other suffer from mental illness?
9. Were you separated from one of your parents for more than 1 year?
10. Was either of your parents unemployed for more than 1 year when they wanted to be working?
11. Was parental, sibling, or significant other alcohol or drug use severe enough to cause family problems?
12. Did parents, siblings, or significant others engage in criminal activities severe enough to cause significant stress or worry?
13. Did you experience any other major upheaval that you think may have shaped your life or personality significantly?

Supplement III: Participant safety considerations

We only recruited participants aged 18 and over, as due to the childhood adversity measure otherwise additional risk assessments would have had to be available for under 18-year olds, which would have been difficult to realize. Realistically, the number of under 18-year old students was also negligible. Moreover, we asked broadly about childhood adversity, but did not ask about event-related details. This did not give enough information which would have identified a participant at risk of current adversity. Our mental health outcome is the GHQ-12 (e.g. Hankins, 2008), which measures general mental health and wellbeing, and does neither act as a screening nor as a diagnostic tool for diagnosable mental disorders. Therefore, we would not have been able to identify people with (risk of) diagnosable mental illness(es). However, we put details of how to get help and support in case the study would bring up difficult feelings, or in case a participant would want to report child maltreatment/ a crime, into a mental health services information sheet, which could be downloaded from the online questionnaire.

Supplement IV: Latent Growth Models with varying residual variances

Latent Growth Model Fit

	AIC	BIC	CFI	TLI	RMSEA	RMSEA CI	SRMR	Chi^2 vs M1 (df)	p
Perceived stress									
M1	4718.05	4746.83	0.98	0.96	0.07	0.01-0.13	0.04	-	-
**M1****	**4716.06**	**4740.73**	**0.98**	**0.98**	**0.05**	**0.00-0.11**	**0.04**	**-**	**-**
M1*	4718.06	4746.84	0.98	0.96	0.07	0.01-0.13	0.04	-	**-**
M1**a	4718.01	4746.79	0.98	0.96	0.07	0.01-0.13	0.04	00.06 (1)[†1]	.81
M1**b	4716.00	4744.78	0.99	0.98	0.05	0.00-0.12	0.03	01.65 (1)[†1]	.20
M1**c	4715.78	4744.56	0.99	0.98	0.05	0.00-0.12	0.05	02.30 (1)[†1]	.13
M2	4811.92	4836.59	0.47	0.47	0.27	0.23-0.32	0.18	-	-
Mental distress									
M1	6295.92	6324.66	0.94	0.91	0.09	0.04-0.15	0.05	-	-
M1a	6296.78	6329.63	0.94	0.82	0.13	0.06-0.21	0.04	01.14 (1)	.29
**M1b**	**6289.60**	**6322.45**	**1.00**	**1.00**	**0.00**	**0.00-0.12**	**0.02**	**10.48 (1)**	**< .01**
M1c	6294.13	6326.99	0.96	0.89	0.10	0.03-0.19	0.05	02.41 (1)	.12
M2	6366.44	6391.08	0.32	0.32	0.24	0.20-0.29	0.19	-	-
M2**	6403.08	6423.61	0.00	0.25	0.26	0.22-0.30	0.19	-	-
**M2***	**6364.52**	**6389.17**	**0.34**	**0.34**	**0.24**	**0.20-0.29**	**0.18**	**-**	**-**
M2*b	6366.11	6394.86	0.34	0.00	0.29	0.24-0.35	0.18	00.38 (1)[†2]	.54

Note. AIC = Akaike information criterion, BIC = Bayesian information criterion, CFI = confirmatory fit index, TLI = Tucker-Lewis fit index, RMSEA = root mean square error of approximation, CI = confidence interval, SRMR = standardized root mean square residual, df = degrees of freedom, p = p-value. M1 = freely estimated trajectory with invariant residual variances. M1* = the same model as M1 but this time with the variance for the latent slope constrained to >0, to render it non-negative. M1** = the same model as M1 but this time with the variance for the latent slope fixed to 0, to render it non-negative. M1a-c = freely estimated trajectory with a varying residual variance for one of the three occasions. M1a = freed residual variance for occasion 1. M1b = freed residual variance for occasion 2. M1c = freed residual variance for occasion 3. M2 = no-change trajectory model with invariant residual variances. M2* = the same model as M2 but this time with the variance for the latent slope constrained to >0, to render it non-negative. M2** = the same model as M2 but this time with the variance for the latent slope fixed to 0, to render it non-negative. M2*b = the same model as M2* but this time with a varying residual variance for occasion 2. [†1]Models M1**a-c have been compared against model M1**. [†2]Model M2*b has been compared against model M2*.

Supplement V: Demographic and clinical characteristics for the sample with data for at least 2 occasions

Demographic and Clinical Statistics, for the Overall Sample with Data for at least Two Occasions (N = 324)

	**Percentages % and Sample Sizes (*N*) per Answer Category**					
**Academic year**	1= 20%(66)	2= 18%(60)	3= 16%(51)	4= 19%(61)	5= 12%(38)	6= 15%(48)
**Gender***	female = 61%(197)		male = 37%(121)		prefer not to say = 2%(5)	
**Age****	18-20 = 37%(121)	21-23 = 43%(140)	24-26 = 14%(45)	27-29 = 3%(11)	30+ = 1%(4)	Median = 2 (age 21-23), IQR = 1
**Ethnicity *****	white = 61%(199)			non-white = 37%(121)		
**Therapeutic treatment**	no = 86%(279) [1]			yes = 14%(45) [1]		
**Psychopharmaceutic treatment**	no = 88%(284) [1]			yes = 12%(40) [1]		
**Education** (further/ higher education after secondary school)	Mother:<br>Father*:	yes=80%(258)<br>yes=82%(266)		no=20%(64)<br>no=17%(55)		unknown=0%(2)<br>unknown=1%(2)

Note. IQR = inter quartile range. [1]treatment for the 6 months prior to occasion 1. *1 participant has not provided information. **3 participants did not provide information. ***4 participants did not provide information. Due to the missingness, some percentages do not add up.

(Di)stress Levels for the Three Occasions, for the Sample with Data for at least Two Occasions (N = 324)

	**Occasion 1 M(SD)**	**Occasion 2 M(SD)**	**Occasion 3 M(SD)**
PSS	10.29(2.71), N = 324	11.61(2.77), N = 274	9.89(2.67), N = 282
GHQ-12	25.13(5.59), N = 320	27.39(6.09), N = 273	23.31(5.93), N = 282

Note. M = mean, SD = standard deviation, N = sample size. PSS = Perceived Stress Scale, GHQ-12 = General Health Questionnaire, 12 item version.

Supplement VI: Missingness predictors and analyses results when including the auxiliary variables

Missingness Predictors for the Analyses Variables

	**Perceived stress o1**	**Perceived stress o2**	**Perceived stress o3**	**Distress o1**	**Distress o2**	**Distress o3**
Missing responses	0	177	169	6	178	169
Perceived stress o1	-	0.10†	0.31	0.87	0.12	0.25
Perceived stress o2	-	-	0.39	0.94	0.63	0.44
Perceived stress o3	-	0.98	-	0.48	0.83	-
**Global stress slider o1**	**-**	**<0.05***	**0.33**	**0.51**	**<0.05***	**0.26**
Global stress slider o2	-	-	0.91	0.57	0.58	0.78
Global stress slider o3	-	0.21	-	0.78	0.10	-
Mental distress o1	-	0.12	0.45	-	0.12	0.38
Mental distress o2	-	-	0.92	0.75	-	0.85
Mental distress o3	-	0.51	-	0.69	0.43	-
Psychotherapy o1	-	1	0.34	0.71	1	0.50
Psychotherapy o2	-	-	0.12	1	1	0.33
Psychotherapy o3	-	0.30	-	1	0.13	-
**Medication use o1**	**-**	**0.08†**	**0.07†**	**0.84**	**<0.05***	**0.13**
Medication use o2	-	-	0.57	1	1	0.94
Medication use o3	-	0.35	-	0.97	0.32	-
**Gender**	**-**	**0.07†**	**0.06†**	**1**	**<0.05***	**0.09†**
**Ethnicity**	**-**	**<0.01****	**0.06†**	**1**	**<0.01****	**<0.05***
Academic year	-	0.90	0.32	0.33	0.83	0.25
Age	-	0.99	0.42	0.69	0.92	0.42
Retrospectively (o3) reported exam stress	-	0.09†	-	0.91	0.19	-
Adversity	-	0.26	0.87	0.60	0.38	0.85
Adversity (binary)	-	0.11	0.38	1	0.17	0.38

Note. Missing responses counts the number of missing responses out of the 451 possible responses. O = occasion. All other depicted values represent *p* values that describe the (non)significance with which the variables predict missingness of the analyses variables. For binary predictors we conducted Pearson's Chi-squared tests with Yates' continuity correction and for continuous predictors Wilcoxon rank sum tests with continuity correction. Some tests could not successfully be conducted, as there either was too little missingness or too much overlapping missingness, which is indicated with "-". All analyses variables can be considered continuous. All variables are described in detail in the main manuscript. Medication use is limited to psychopharmacological medication.

Latent Growth Model Summary – With Auxiliary Variables

	**Slope loa-ding o1**	**Slope loa-ding o2**	**Slope loa-ding o3**	**Intercept mean**	**Slope mean**	**Residual var. o1**	**Residual var. o2**	**Residual var. o3**	**Intercept slope covar.**
Perceived stress									
M1	0.28	1.00	0.00	09.92	01.79	3.85	3.85	3.85	0.57
**M1***	**0.29**	**1.00**	**0.00**	**09.91**	**01.79**	**3.77**	**3.77**	**3.77**	**0.45**
M2	0.53	1.00	0.00	10.53	00.00	3.73	3.73	3.73	-1.25
Mental distress									
**M1**	**0.59**	**1.00**	**0.00**	**23.21**	**04.14**	**17.03**	**17.03**	**17.03**	**-8.08**
M2	-4.33	1.00	0.00	25.46	00.00	37.36	37.36	37.36	-1.64
M2*	0.67	1.00	0.00	25.27	00.00	16.80	16.80	16.80	-17.97

Note. o = occasion, var. = variance, covar. = covariance; M1 = the freely estimated trajectory model. M1* = the M1 model but this time with the variance for the latent slope constrained to >0, to render it non-negative. M2 = the no-change trajectory model. M2* = the M2 model but this time with the variance for the latent slope constrained to >0, to render it non-negative.

Latent Growth Model Fit – with Auxiliary Variables

	AIC	BIC	CFI	TLI	RMSEA	SRMR	Chi² (df)	Chi² vs M1 (df)	p
Perceived stress									
M1	6386.72	6522.40	0.98	0.96	0.07	0.02	6.46 (2)	-	-
**M1***	**6386.82**	**6522.50**	**0.98**	**0.96**	**0.07**	**0.02**	**6.56 (2)**	**-**	**-**
M2	6479.41	6610.98	0.47	0.47	0.27	0.09	101.14 (3)	74.37 (1)	< .001
Mental distress									
**M1**	**7998.99**	**8134.67**	**0.93**	**0.90**	**0.09**	**0.03**	**9.92 (2)**	**-**	**-**
M2	8069.87	8201.43	0.31	0.31	0.24	0.10	82.79 (3)	62.19 (1)	< .001
M2*	8068.00	8199.56	0.33	0.33	0.24	0.09	80.92 (3)	57.90 (1)	< .001

Note. AIC = Akaike information criterion, BIC = Bayesian information criterion, CFI = confirmatory fit index, TLI = Tucker-Lewis fit index, RMSEA = root mean square error of approximation, SRMR = standardized root mean square residual, df = degrees of freedom, p = p-value. M1 = the freely estimated trajectory model. M1* = the M1 model but this time with the variance for the latent slope constrained to >0, to render it non-negative. M2 = the no-change trajectory model. M2* = the M2 model but this time with the variance for the latent slope constrained to >0, to render it non-negative.

Bivariate Latent Change Score Models Summary: Occasion 1 to 2 – with Auxiliary Variables

	coefficient	SE	standardized coefficient	z-value	p-value
**(auto)regression**					
stress o1 → distress c(o1-o2)	0.250	0.160	0.122	1.562	0.118
distress o1 → distress c(o1-o2)	-0.509	0.087	-0.519	-5.841	0.000
distress o1 → stress c(o1-o2)	0.144	0.041	0.331	3.527	0.000
stress o1 → stress c(o1-o2)	-0.635	0.083	-0.694	-7.644	0.000
**covariances**					
stress o1 ←→ distress o1	11.849	0.965	0.737	12.280	0.000
stress c(o1-o2) ←→ distress c(o1-o2)	6.622	0.841	0.591	7.875	0.000
**intercepts**					
stress c(o1-o2)	4.258	0.627	1.681	6.786	0.000
stress o1	10.424	0.130	3.762	79.895	0.000
distress c(o1-o2)	12.566	1.586	2.208	7.925	0.000
distress o1	25.399	0.274	4.375	92.703	0.000
**variances**					
stress c(o1-o2)	4.795	0.443	0.747	10.820	0.000
stress o1	7.677	0.491	1.000	15.649	0.000
distress c(o1-o2)	26.208	2.284	0.809	11.476	0.000
distress o1	33.702	2.636	1.000	12.788	0.000

Note. SE = standard error. O = occasion.

Bivariate Latent Change Score Models Summary: Occasion 1 to 3 – with Auxiliary Variables

	coefficient	SE	standardized coefficient	z-value	p-value
**(auto)regression**					
stress o1 → distress c(o1-o3)	0.358	0.199	0.144	1.805	0.071
distress o1 → distress c(o1-o3)	-0.806	0.097	-0.679	-8.342	0.000
distress o1 → stress c(o1-o3)	-0.025	0.038	-0.050	-0.648	0.517
stress o1 → stress c(o1-o3)	-0.534	0.086	-0.514	-6.231	0.000
**covariances**					
stress o1 ←→ distress o1	11.848	0.964	0.737	12.286	0.000
stress c(o1-o3) ←→ distress c(o1-o3)	8.269	1.121	0.615	7.375	0.000
**Intercepts**					
stress c(o1-o3)	5.702	0.680	1.982	8.381	0.000
stress o1	10.424	0.130	3.762	79.895	0.000

distress c(o1-o3)	14.756	1.699	2.142	8.685	0.000
distress o1	25.401	0.274	4.375	92.669	0.000
**variances**					
stress c(o1-o3)	5.755	0.549	0.695	10.479	0.000
stress o1	7.677	0.491	1.000	15.649	0.000
distress c(o1-o3)	31.412	2.837	0.662	11.074	0.000
distress o1	33.705	2.635	1.000	12.791	0.000

Note. SE = standard error. O = occasion.

Bivariate Latent Change Score Models Summary: Occasion 2 to 3 – with Auxiliary Variables

	coefficient	SE	standardized coefficient	z-value	p-value
**(auto)regression**					
stress o2 → distress c(o2-o3)	0.479	0.194	0.178	2.471	0.013
distress o2 → distress c(o2-o3)	-0.925	0.101	-0.758	-9.178	0.000
distress o2 → stress c(o2-o3)	-0.021	0.035	-0.045	-0.611	0.541
stress o2 → stress c(o2-o3)	-0.544	0.077	-0.525	-7.059	0.000
**covariances**					
stress o2 ←→ distress o2	11.899	1.097	0.709	10.851	0.000
stress c(o2-o3) ←→ distress c(o2-o3)	8.244	1.003	0.612	8.217	0.000
**intercepts**					
stress c(o2-o3)	5.129	0.731	1.793	7.021	0.000
stress o2	11.679	0.160	4.232	72.839	0.000
distress c(o2-o3)	15.664	1.870	2.112	8.376	0.000
distress o2	27.542	0.370	4.530	74.460	0.000
**variances**					
stress c(o2-o3)	5.643	0.461	0.689	12.250	0.000
stress o2	7.616	0.633	1.000	12.025	0.000
distress c(o2-o3)	32.202	2.892	0.585	11.136	0.000
distress o2	36.967	3.057	1.000	12.093	0.000

Note. SE = standard error. O = occasion.

Supplement VII: Analyses results when excluding one potentially influential case

Latent Growth Model Summary – Exclusion of One Potentially Influential Case

	Slope loa-ding o1	Slope loa-ding o2	Slope loa-ding o3	Intercept mean	Slope mean	Residual var. o1	Residual var. o2	Residual var. o3	Intercept slope covar.
Perceived stress									
**M1**	**0.32**	**1.00**	**0.00**	**09.87**	**01.82**	**3.51**	**3.51**	**3.51**	**0.22**
M2	0.49	1.00	0.00	10.53	00.00	3.45	3.45	3.45	-1.51
Mental distress									
**M1**	**0.59**	**1.00**	**0.00**	**23.16**	**04.16**	**16.40**	**16.40**	**16.40**	**-8.27**
M2	-5.26	1.00	0.00	25.43	00.00	37.36	37.36	37.36	-1.58
M2*	0.65	1.00	0.00	25.22	00.00	16.33	16.33	16.33	-17.79

Note. o = occasion, var. = variance, covar. = covariance; M1 = the freely estimated trajectory model. M2 = the no-change trajectory model. M2* = the M2 model but this time with the variance for the latent slope constrained to >0, to render it non-negative.

Latent Growth Model Fit – Exclusion of One Potentially Influential Case

	AIC	BIC	CFI	TLI	RMSEA	SRMR	Chi^2 (df)	Chi^2 vs M1 (df)	p
Perceived stress									
**M1**	**4684.64**	**4713.40**	**0.98**	**0.97**	**0.07**	**0.04**	**6.00 (2)**	**-**	**-**
M2	4779.49	4804.14	0.49	0.49	0.27	0.18	102.85 (3)	98.62 (1)	< .001
Mental distress									
**M1**	**6264.12**	**6292.85**	**0.96**	**0.93**	**0.08**	**0.04**	**7.35 (2)**	**-**	**-**
M2	6338.19	6362.82	0.33	0.33	0.25	0.20	83.42 (3)	62.52 (1)	< .001
M2*	6336.29	6360.91	0.34	0.34	0.24	0.19	81.51 (3)	61.97 (1)	< .001

Note. AIC = Akaike information criterion, BIC = Bayesian information criterion, CFI = confirmatory fit index, TLI = Tucker-Lewis fit index, RMSEA = root mean square error of approximation, SRMR = standardized root mean square residual, df = degrees of freedom, p = p-value. M1 = the freely estimated trajectory model. M2 = the no-change trajectory model. M2* = the M2 model but this time with the variance for the latent slope constrained to >0, to render it non-negative.

Bivariate Latent Change Score Models Summary: Occasion 1 to 2 – Exclusion of One Potentially Influential Case

	coefficient	SE	standardized coefficient	z-value	p-value
**(auto)regression**					
stress o1 → distress c(o1-o2)	0.231	0.158	0.112	1.463	0.144
distress o1 → distress c(o1-o2)	-0.499	0.088	-0.510	-5.675	0.000
distress o1 → stress c(o1-o2)	0.147	0.040	0.341	3.632	0.000
stress o1 → stress c(o1-o2)	-0.627	0.082	-0.692	-7.636	0.000
**covariances**					
stress o1 ↔ distress o1	11.828	0.967	0.736	12.236	0.000
stress c(o1-o2) ↔ distress c(o1-o2)	6.532	0.844	0.587	7.741	0.000
**intercepts**					
stress c(o1-o2)	4.096	0.621	1.635	6.598	0.000
stress o1	10.433	0.130	3.772	80.020	0.000
distress c(o1-o2)	12.426	1.607	2.187	7.730	0.000
distress o1	25.410	0.275	4.375	92.524	0.000
**variances**					
stress c(o1-o2)	4.722	0.440	0.752	10.729	0.000
stress o1	7.650	0.491	1.000	15.592	0.000
distress c(o1-o2)	26.211	2.305	0.812	11.371	0.000
distress o1	33.737	2.645	1.000	12.754	0.000

Note. SE = standard error. O = occasion.

Bivariate Latent Change Score Models Summary: Occasion 1 to 3 – Exclusion of One Potentially Influential Case

	coefficient	SE	standardized coefficient	z-value	p-value
**(auto)regression**					
stress o1 → distress c(o1-o3)	0.426	0.195	0.175	2.184	0.029
distress o1 → distress c(o1-o3)	-0.810	0.097	-0.699	-8.378	0.000
distress o1 → stress c(o1-o3)	-0.025	0.038	-0.053	-0.653	0.514
stress o1 → stress c(o1-o3)	-0.496	0.085	-0.497	-5.824	0.000
**covariances**					
stress o1 ←→ distress o1	11.822	0.966	0.736	12.240	0.000
stress c(o1-o3) ←→ distress c(o1-o3)	7.554	0.884	0.593	8.544	0.000
**Intercepts**					
stress c(o1-o3)	5.288	0.632	1.917	8.363	0.000
stress o1	10.433	0.130	3.772	80.020	0.000
distress c(o1-o3)	14.089	1.589	2.092	8.868	0.000
distress o1	25.413	0.275	4.377	92.519	0.000
**variances**					
stress c(o1-o3)	5.410	0.436	0.711	12.421	0.000
stress o1	7.650	0.491	1.000	15.592	0.000
distress c(o1-o3)	30.003	2.501	0.661	11.998	0.000
distress o1	33.714	2.643	1.000	12.757	0.000

Note. SE = standard error. O = occasion.

Bivariate Latent Change Score Models Summary: Occasion 2 to 3 – Exclusion of One Potentially Influential Case

	coefficient	SE	standardized coefficient	z-value	p-value
**(auto)regression**					
stress o2 → distress c(o2-o3)	0.451	0.199	0.169	2.272	0.023
distress o2 → distress c(o2-o3)	-0.923	0.102	-0.761	-9.080	0.000
distress o2 → stress c(o2-o3)	-0.022	0.035	-0.046	-0.619	0.536
stress o2 → stress c(o2-o3)	-0.549	0.079	-0.534	-6.979	0.000
**covariances**					
stress o2 ←→ distress o2	12.034	1.122	0.710	10.722	0.000
stress c(o2-o3) ←→ distress c(o2-o3)	7.961	0.943	0.603	8.445	0.000
**intercepts**					
stress c(o2-o3)	5.180	0.743	1.815	6.970	0.000
stress o2	11.630	0.165	4.188	70.424	0.000
distress c(o2-o3)	15.896	1.903	2.146	8.354	0.000
distress o2	27.401	0.366	4.489	74.922	0.000
**variances**					
stress c(o2-o3)	5.518	0.440	0.678	12.547	0.000
stress o2	7.713	0.659	1.000	11.697	0.000
distress c(o2-o3)	31.553	2.830	0.575	11.148	0.000
distress o2	37.261	3.164	1.000	11.777	0.000

Note. SE = standard error. O = occasion.

Supplement VIII: Model summaries and exact coefficients of the Bivariate Latent Change Score Models

Bivariate Latent Change Score Models Summary: Occasion 1 to 2

	coefficient	SE	standardized coefficient	z-value	p-value
**(auto)regression**					
stress o1 → distress c(o1-o2)	0.219	0.158	0.106	1.384	0.166
distress o1 → distress c(o1-o2)	-0.499	0.088	-0.509	-5.676	0.000
distress o1 → stress c(o1-o2)	0.148	0.040	0.338	3.647	0.000
stress o1 → stress c(o1-o2)	-0.638	0.083	-0.698	-7.722	0.000
**covariances**					
stress o1 ←→ distress o1	11.865	0.967	0.737	12.274	0.000
stress c(o1-o2) ←→ distress c(o1-o2)	6.608	0.841	0.590	7.860	0.000
**intercepts**					
stress c(o1-o2)	4.212	0.627	1.663	6.722	0.000
stress o1	10.424	0.130	3.762	79.895	0.000
distress c(o1-o2)	12.561	1.602	2.207	7.843	0.000
distress o1	25.396	0.274	4.371	92.555	0.000
**variances**					
stress c(o1-o2)	4.791	0.443	0.747	10.821	0.000
stress o1	7.677	0.491	1.000	15.649	0.000
distress c(o1-o2)	26.230	2.289	0.810	11.458	0.000
distress o1	33.753	2.643	1.000	12.770	0.000

Note. SE = standard error. O = occasion.

Bivariate Latent Change Score Models Summary: Occasion 1 to 3

	coefficient	SE	standardized coefficient	z-value	p-value
**(auto)regression**					
stress o1 → distress c(o1-o3)	0.376	0.200	0.152	1.879	0.060
distress o1 → distress c(o1-o3)	-0.808	0.096	-0.682	-8.382	0.000
distress o1 → stress c(o1-o3)	-0.024	0.038	-0.048	-0.617	0.537
stress o1 → stress c(o1-o3)	-0.520	0.088	-0.505	-5.898	0.000
**covariances**					
stress o1 ←→ distress o1	11.859	0.966	0.737	12.279	0.000
stress c(o1-o3) ←→ distress c(o1-o3)	8.267	1.118	0.615	7.392	0.000
**intercepts**					
stress c(o1-o3)	5.541	0.674	1.942	8.221	0.000
stress o1	10.424	0.130	3.762	79.895	0.000
distress c(o1-o3)	14.604	1.653	2.124	8.835	0.000
distress o1	25.399	0.274	4.373	92.547	0.000
**variances**					
stress c(o1-o3)	5.753	0.549	0.707	10.476	0.000
stress o1	7.677	0.491	1.000	15.649	0.000
distress c(o1-o3)	31.401	2.832	0.664	11.088	0.000
distress o1	33.730	2.641	1.000	12.773	0.000

Note. SE = standard error. O = occasion.

Bivariate Latent Change Score Models Summary: Occasion 2 to 3

	coefficient	SE	standardized coefficient	z-value	p-value
**(auto)regression**					
stress o2 → distress c(o2-o3)	0.477	0.198	0.177	2.409	0.016
distress o2 → distress c(o2-o3)	-0.931	0.101	-0.760	-9.213	0.000
distress o2 → stress c(o2-o3)	-0.025	0.035	-0.053	-0.714	0.475
stress o2 → stress c(o2-o3)	-0.537	0.079	-0.519	-6.834	0.000
**covariances**					
stress o2 ←→ distress o2	11.987	1.116	0.709	10.737	0.000
stress c(o2-o3) ←→ distress c(o2-o3)	8.368	1.020	0.616	8.204	0.000
**intercepts**					
stress c(o2-o3)	5.161	0.741	1.797	6.962	0.000
stress o2	11.636	0.165	4.195	70.687	0.000
distress c(o2-o3)	15.859	1.897	2.125	8.359	0.000
distress o2	27.402	0.364	4.499	75.220	0.000
**variances**					
stress c(o2-o3)	5.685	0.467	0.689	12.165	0.000
stress o2	7.696	0.656	1.000	11.736	0.000
distress c(o2-o3)	32.446	2.941	0.583	11.031	0.000
distress o2	37.103	3.152	1.000	11.771	0.000

Note. SE = standard error. O = occasion.
